# Supplementary material for: Downregulation of Ribosomal Contents and Kinase Activities Is Associated with the Inhibitive Effect on the Growth of Group B Streptococcus Induced by Placental Extracellular Vesicles
Source: Biology (Basel). 2021 Jul 14;10(7):664. doi: 10.3390/biology10070664 (PMC8301483; doi:10.3390/biology10070664)
Supplement: Supplementary file 1 [file biology-10-00664-s001.zip › biology-1257757-supplementary.pdf]

A

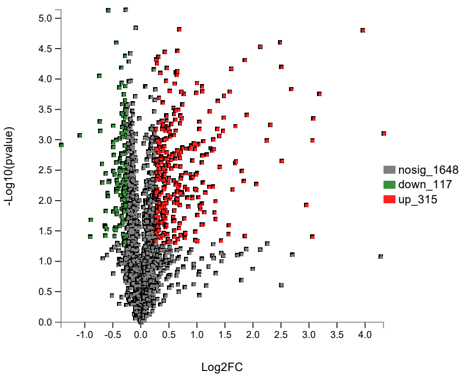

B

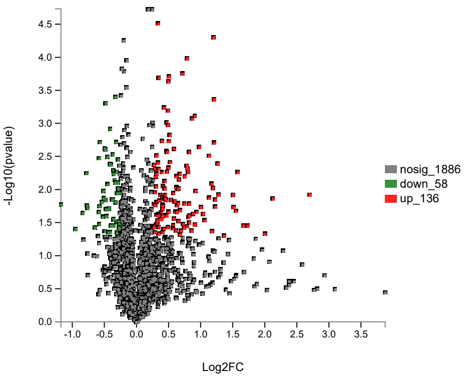

Supplementary figure S1. Summary of quantitative proteomic analysis of GBS treated with placental micro-EVs and nano-EVs. (A) A total of 432 out of 2080 proteins were found to be significantly upregulated/downregulated in placental micro-EVs treated samples compared with controls. (B) A total of 194 out of 2080 proteins were found to be significantly upregulated/downregulated in placental nano-EVs treated samples compared with controls. Proteins with fold changes of >1.20 or <0.83 relative to control and a two-tailed *p*-values lower than 0.05 were considered significantly upregulated or downregulated, respectively.

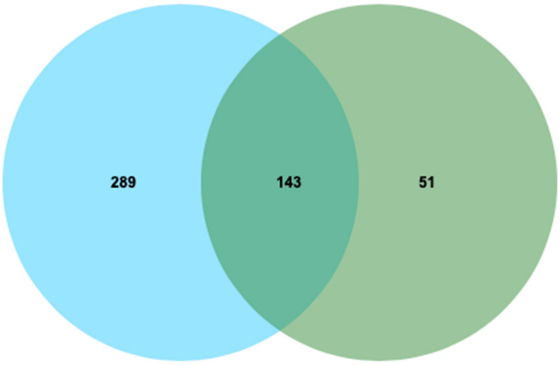

Figure S2. The Venn diagram showed that 143 overlapping proteins were sensitive to both placental micro-and nano-extracellular vesicles treatment. 289 proteins were only detected in GBS with placental micro-EVs treatment (blue circle), and 51 proteins were only found in GBS with placental nano-EVs treatment (green circle).

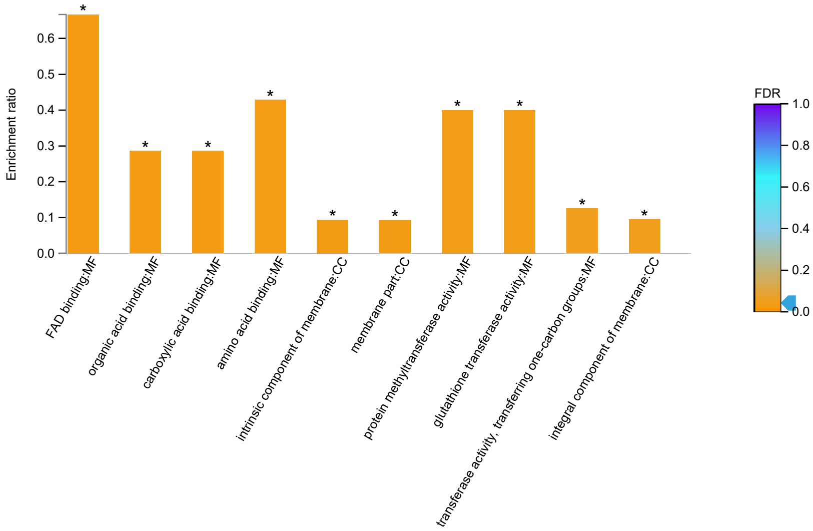

Figure S3. GO enrichment analysis of overlapping proteins

Protein Number	GO ID	GO Description	GO Term(Le	Ratio_in_st	Ratio_in_p	Pvalue_unc	Pvalue_corrected
2	GO:0071949	FAD binding	MF	2/116	3/1804	0.011785	0.032409
4	GO:0043177	organic acid binding	MF	4/116	14/1804	0.009821	0.03241
4	GO:0031406	carboxylic acid binding	MF	4/116	14/1804	0.009821	0.03241
3	GO:0016597	amino acid binding	MF	3/116	7/1804	0.007495	0.035334
39	GO:0031224	intrinsic component of membrane	CC	39/116	413/1804	0.006	0.039603
41	GO:0044425	membrane part	CC	41/116	443/1804	0.007218	0.039698
2	GO:0008276	protein methyltransferase activity	MF	2/116	5/1804	0.036064	0.042504
2	GO:0004364	glutathione transferase activity	MF	2/116	5/1804	0.036064	0.042504
9	GO:0016741	transferase activity, transferring one-carbon groups	MF	9/116	72/1804	0.045237	0.048155
39	GO:0016021	integral component of membrane	CC	39/116	412/1804	0.005893	0.048613

Accession	Regulation	Functional Description
FAD/organic acid/carboxylic acid/amino acid binding		
A0A380J025	up	UDP-N-acetylenolpyruvoylglucosamine reductase
S9BXK9	up	UDP-N-acetylenolpyruvoylglucosamine reductase
A0A0G2ZC45	down	Arginine repressor, transcription factor activity
A0A0H1Z5K9	down	Ornithine carbamoyltransferase , arginine catabolic process to ornithine
A0A656FZZ3	up	Aspartate carbamoyltransferase
B2NHM1	up	Penicillin-binding protein 2x
Transferase		
A0A0E1EN12	up	Fe-S-oxidoreductase
A0A0G2Z569	up	Glutathione S-transferase, omega
A0A0H1Z5K9	down	Ornithine carbamoyltransferase
A0A4U3JA57	up	Cytosine-specific methyltransferase
A0A5N0LJY8	up	RNA methyltransferase
A0A656FZZ3	up	Aspartate carbamoyltransferase
Q8E1T6	down	Uncharacterized protein
Q8E4H7	up	tRNA (guanine-N(1)-)-methyltransferase
S8FQZ0	up	GST C-terminal domain-containing protein
V6Z143	up	Ribosomal protein L11 methyltransferase
V6Z397	up	rRNA methyltransferase
Intrinsic/integral component of membrane		
A0A076YYX7	up	Uncharacterized protein
A0A0E1EGN1	up	ABC transporter permease
A0A0E1EJX5	up	Membrane protein, putative
A0A0E1EQI0	up	Conjugal transfer protein
A0A0G2YZ44	down	Putative zinc transport system permease protein AdcB
A0A0G2Z171	down	Putative deoxyribose-specific ABC transporter, permease protein
A0A0G2Z2W6	down	Cell surface hydrolase (Putative)
A0A0G2Z3U2	up	Two-component sensor histidine kinase, malate
A0A0H1DGL2	up	ClC family H(+)/Cl(-) exchange transporter
A0A0H1GGJ2	up	DUF1310 domain-containing protein
A0A0H1L4V8	down	PTS beta-glucoside transporter subunit IIBCA
A0A0H1QEN8	down	PTS ascorbate transporter subunit IIC
A0A1A9E314	up	Cytochrome C oxidase assembly protein
A0A380IIU7	down	Phage infection protein
A0A380IJ26	up	Quinolone resistance protein NorA
A0A380IM47	up	Pullulanase
A0A3D8VX54	up	PTS galactitol transporter subunit IIC
A0A3P1B8D9	down	DUF4366 domain-containing protein
A0A656G179	down	PTS system sucrose-specific IIBC component
A0A6A4TPK7	up	ABC transporter permease subunit
B2NHM1	up	Penicillin-binding protein 2X
B9UQA9	up	PI-1 ancillary protein 2
Q8E1T6	down	Uncharacterized protein
Q8E3L7	up	Uncharacterized protein
Q8E452	up	MFS domain-containing protein
Q8E5T8	down	Uncharacterized protein
Q8E714	down	Acyl_transf_3 domain-containing protein
R4Z8N4	down	Membrane protein
S4WCS4	up	Uncharacterized protein
S8FJZ6	up	Membrane protein
S8GHL0	up	Cytochrome D ubiquinol oxidase subunit I
S8GJD9	up	Uncharacterized protein
S8H257	up	Conjugal transfer protein TraG
S9AP88	up	Teicoplanin resistance protein VanZ
S9B205	down	Colicin V production protein
V6Z0J7	up	Uncharacterized protein
V6Z0S3	down	Ribose ABC transporter permease
V6Z3L9	up	Sodium:hydrogen antiporter
V6Z3U1	down	Membrane protein
V6Z4Z9	up	ABC transporter substrate-binding protein
A0A0H1WTH4	down	Amino acid ABC transporter permease

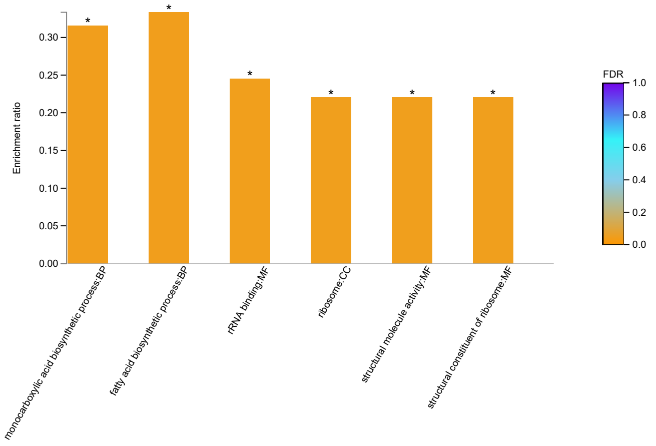

Figure S4. GO enrichment analysis of proteins only detected in GBS with placental micro-EVs treatment

Protein Number	GO ID	GO Description	GO Term(Level1)	Pvalue_corrected
6	GO:0006633	fatty acid biosynthetic process	BP	0.047066015
6	GO:0072330	monocarboxylic acid biosynthetic	BP	0.046917581
15	GO:0005840	ribosome	CC	0.049792414
13	GO:0019843	rRNA binding	MF	0.048303332
15	GO:0005198	structural molecule activity	MF	0.049792414
15	GO:0003735	structural constituent of ribosome	MF	0.049792414

Protein Accession	Regualtion	Functional Description
V6Z6Z4	down	Uncharacterized protein
S8FH53	up	Biotin carboxyl carrier protein of acetyl-CoA carboxylase
Q8E3E6	down	30S ribosomal protein S7
S8FKP1	up	30S ribosomal protein S15
A0A5N0R7P5	up	Ribonuclease M5
S9B2D4	up	3-oxoacyl-[acyl-carrier-protein] synthase 2
A0A656G1D0	up	4'-phosphopantetheinyl transferase5
V6YZ80	down	30S ribosomal protein S13
A0A0G9JEY8	up	3-oxoacyl-[acyl-carrier-protein] synthase 2
V6Z275	down	S1 RNA-binding protein
Q3K0C9	down	50S ribosomal protein L35
V6Z128	down	30S ribosomal protein S9
R4Z934	down	30S ribosomal protein S11
A0A1C0BEJ0	down	50S ribosomal protein L21
Q8DYG1	down	50S ribosomal protein L11
S9C983	up	50S ribosomal protein L23
A0A6A4TZH8	down	50S ribosomal protein L3
Q8DZZ2	down	30S ribosomal protein S20
V6YZ47	down	50S ribosomal protein L3
V6Z0L9	down	50S ribosomal protein L10
V6Z1H0	down	Biotin carboxyl carrier protein of acetyl-CoA carboxylase
V6Z135	down	30S ribosomal protein S11
V6Z2C6	down	50S ribosomal protein L7/L12
Q8E5S2	up	Uncharacterized protein

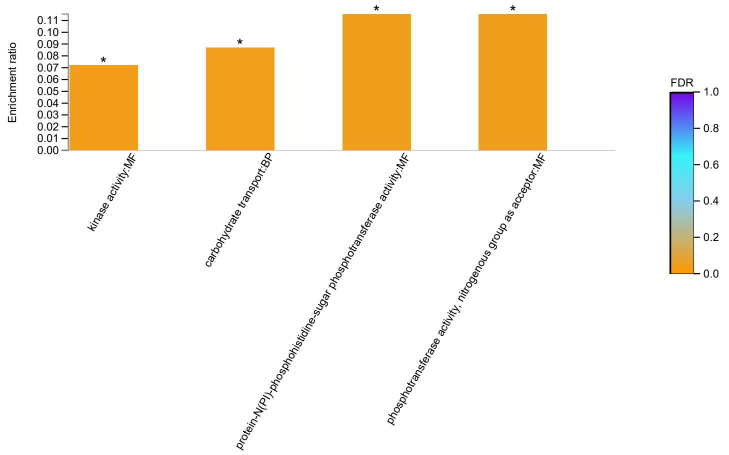

Figure S5. GO enrichment analysis of proteins only detected in GBS with placental nano-EVs treatment

Protein Number	GO ID	GO Description	GO Term(Level1)	Pvalue_corrected
4	GO:0008643	carbohydrate transport	BP	0.048242083
9	GO:0016301	kinase activity	MF	0.044508353
3	GO:0008982	protein-N(PI)-phosphohistidine-sugar phosphotransferase activity	MF	0.049870836
3	GO:0016775	phosphotransferase activity, nitrogenous group as acceptor	MF	0.049870836

Protein Accession	Regualtion	Functional Description
A0A0H1NG81	down	Putative galactitol operon regulator (Transcriptional antiterminator), BglG family PTS system, mannitolfructose-specific IIA component
V6Z7B9	down	Lipid kinase
Q8E2P0	down	Uncharacterized protein
A0A0H1U8Y4	down	Glycerol kinase
P65204	down	Adenylate kinase
S9B0Q5	down	PTS galactitol transporter subunit IIC
V6Z5I1	up	PTS mannose transporter subunit IIAB
A0A380IM67	up	Two-component sensor histidine kinase
A0A380IY32	down	PTS system, sucrose-specific IIB component / PTS system, sucrose-specific IIC component / PTS system, sucrose-specific IIA component
Q8DX04	down	Sensor histidine kinase
V6Z3K8	down	Thymidylate kinase
